# Supplementary material for: Examining specific emotion dynamics in daily life in male adolescents: An experience sampling method study
Source: PLOS Ment Health. 2026 Jan 7;3(1):e0000513. doi: 10.1371/journal.pmen.0000513 (PMC12798519; doi:10.1371/journal.pmen.0000513)
Supplement: S1 Table — (DOCX) [file pmen.0000513.s001.docx]

**Supplementary Table S1**. Estimated marginal means of positive and negative emotions across personal and external environments.

| **Estimations** | | | | | |
| --- | --- | --- | --- | --- | --- |
| Dependent variable | | Mean | Standard error | 95% Confidence interval | |
|  |  |  |  | Lower bound | Upper bound |
| Positive emotions | Personal environment | 71.82 | 1.00 | 69.86 | 73.79 |
|  | External environment | 79.06 | 1.31 | 76.49 | 81.63 |
| Negative emotions | Personal environment | 18.05 | 0.84 | 16.41 | 19.70 |
|  | External environment | 14.63 | 1.09 | 12.48 | 16.77 |
| Positive granularity | Personal environment | -1.09 | 0.04 | -1.17 | -1.00 |
|  | External environment | -1.00 | 0.06 | -1.11 | -0.89 |
| Negative granularity | Personal environment | -1.06 | 0.04 | -1.13 | -0.99 |
|  | External environment | -0.91 | 0.05 | -1.00 | -0.82 |
