# Supplementary material for: Examining specific emotion dynamics in daily life in male adolescents: An experience sampling method study
Source: PLOS Ment Health. 2026 Jan 7;3(1):e0000513. doi: 10.1371/journal.pmen.0000513 (PMC12798519; doi:10.1371/journal.pmen.0000513)
Supplement: S2 Table — (DOCX) [file pmen.0000513.s002.docx]

**Supplementary Table S2.** Estimated marginal means of positive emotion expression across presence types.

| **Estimations** | | | | | |
| --- | --- | --- | --- | --- | --- |
| Dependent variable | | Mean | Standard error | 95% Confidence interval | |
|  |  |  |  | Lower bound | Upper bound |
| Positive emotions | Alone | 75.72 | 1.17 | 73.43 | 78.01 |
|  | Friends or family | 80.74 | 1.05 | 78.68 | 82.79 |
|  | Others | 69.87 | 1.91 | 66.12 | 73.62 |
| Negative emotions | Alone | 15.96 | 0.98 | 14.05 | 17.87 |
|  | Friends or family | 14.28 | 0.87 | 12.57 | 16.00 |
|  | Others | 18.78 | 1.60 | 15.64 | 21.91 |
