# Supplementary material for: Examining specific emotion dynamics in daily life in male adolescents: An experience sampling method study
Source: PLOS Ment Health. 2026 Jan 7;3(1):e0000513. doi: 10.1371/journal.pmen.0000513 (PMC12798519; doi:10.1371/journal.pmen.0000513)
Supplement: S2 Fig — (DOCX) [file pmen.0000513.s004.docx]

**Supplementary Fig S2**. Role of context on emotional granularity

Note. *p≤ .05; ** p ≤ .01

Positive emotions = “good”, “quiet”, “happy”

Negative emotion= “nervous/excited”, “anxious/fear”, “angry/annoyed”

Example of items for Familiar context: “at home in my room”, “at the household in my room”

Example of items for External context: “in a restaurant or bar”, “in a public building”
