# Supplementary material for: Examining specific emotion dynamics in daily life in male adolescents: An experience sampling method study
Source: PLOS Ment Health. 2026 Jan 7;3(1):e0000513. doi: 10.1371/journal.pmen.0000513 (PMC12798519; doi:10.1371/journal.pmen.0000513)
Supplement: S3 Fig — (DOCX) [file pmen.0000513.s005.docx]

**Supplementary Fig S3**. Role of presence of others on intensity of positive and negative emotions

Note. * p <.05, ** p <.01, corrected for least square differences (LSD).

Example of items for Family /friend: “family or partner”, “one or more friends”

Example of items for Alone: “nobody”

Example of items for other: “Colleague or classmates”, “strangers”, “an educator”, “carers”, “a pet”, “other”.
