# Supplementary material for: Examining specific emotion dynamics in daily life in male adolescents: An experience sampling method study
Source: PLOS Ment Health. 2026 Jan 7;3(1):e0000513. doi: 10.1371/journal.pmen.0000513 (PMC12798519; doi:10.1371/journal.pmen.0000513)
Supplement: Attachment — Submitted filename: R2R_270625.docx [file pmen.0000513.s007.docx]

**To the Editor and the Reviewer**

We would like to thank the Editor and the Reviewer for their thoughtful comments on our manuscript. We believe that the changes made to the manuscript based on the Editor’s and Reviewer’s suggestions were important for making this a much stronger and clearer report of our study. In gray are the comments of the Editor and Reviewers, we respond below in normal text and the *text in italics* are part from the manuscript. In the manuscript, the changes are highlighted in yellow.

**Editor**

1. Please provide additional details regarding participant consent. In the ethics statement in the Methods and online submission information, please ensure that you have specified (1) whether consent was informed and (2) what type you obtained (for instance, written or verbal, and if verbal, how it was documented and witnessed). If your study included minors, state whether you obtained consent from parents or guardians. If the need for consent was waived by the ethics committee, please include this information.

**Authors’ response:** This information are now described in 2.2 Ethical approval and consent to participate, where it reads (p.5).

*“The study was authorized by the ethics committee of the Canton of Vaud (#2019-02318). Each adolescent and their legal representative completed a written consent form after receiving oral and written information about the study.”*

If you are reporting a retrospective study of medical records or archived samples, please ensure that you have discussed whether all data were fully anonymized before you accessed them and/or whether the IRB or ethics committee waived the requirement for informed consent. If patients provided informed written consent to have data from their medical records used in research, please include this information.

**Authors’ response:** Not applicable, we have conducted a prospective study.

2. We ask that a manuscript source file is provided at Revision. Please upload your manuscript file as a .doc, .docx, .rtf or .tex.

**Authors’ response:** Accordingly, we uploaded the source file for the Revision.

3. We do not publish any copyright or trademark symbols that usually accompany proprietary names, eg (R), (C), or TM (e.g. next to drug or reagent names). Please remove all instances of trademark/copyright symbols throughout the text, including ® on page 5.

**Authors’ response:** Accordingly, we omitted the symbol ® for Redcap.

4. We note that your Data Availability Statement is currently as follows: [Data is provided within the manuscript. Further inquiries can be directed to the corresponding authors.]

Please confirm at this time whether or not your submission contains all raw data required to replicate the results of your study. Authors must share the “minimal data set” for their submission. PLOS defines the minimal data set to consist of the data required to replicate all study findings reported in the article, as well as related metadata and methods (<https://journals.plos.org/plosone/s/data-availability#loc-minimal-data-set-definition>).

For example, authors should submit the following data:

- The values behind the means, standard deviations and other measures reported;
- The values used to build graphs;

- The points extracted from images for analysis.

Authors do not need to submit their entire data set if only a portion of the data was used in the reported study.

If your submission does not contain these data, please either upload them as Supporting Information files or deposit them to a stable, public repository and provide us with the relevant URLs, DOIs, or accession numbers. For a list of recommended repositories, please see <https://journals.plos.org/plosone/s/recommended-repositories>.

If there are ethical or legal restrictions on sharing a de-identified data set, please explain them in detail (e.g., data contain potentially sensitive information, data are owned by a third-party organization, etc.) and who has imposed them (e.g., an ethics committee). Please also provide contact information for a data access committee, ethics committee, or other institutional body to which data requests may be sent. If data are owned by a third party, please indicate how others may request data access.

**Authors’ response:** Due to ethical reasons (i.e., some participants did not consent on the re-use of their data), all raw data cannot be public available. This has been described in the manuscript. In addition, a supplementary file with more information about the data related to the Figures is provided and the corresponding author is available to answer to specific queries about the data.

**Editor**

Critically address the changes raised by the reviewers.

**Authors’ response:** Below you can find a point-by-point letter addressing each Reviewers’ concerns.

**Reviewer 1**

Major Points:

This study uses an experience sampling method to examine emotion dynamics in male adolescents, but there are several methodological and interpretive concerns that should be addressed before publication.

**Authors’ response:** We would like to thank Reviewer 1 for the thoughtful comments that helped us to enhance the clarity of the manuscript. Below, you can find a point-by-point response to Reviewer 1’s concerns.

The correlation coefficients reported in Tables 2a and 2b include values as small as -0.02 that are marked as statistically significant. While these may clear the p-value threshold due to the large sample size (62 participants × 9 days × 4 measurements = ~2,200+ observations), the practical significance of such tiny correlations is questionable. A correlation of -0.02 indicates essentially no relationship between variables. I strongly recommend that you distinguish between statistical and practical significance throughout the manuscript and consider using effect size interpretations (e.g., Cohen's guidelines where r=.10 is small, r=.30 is medium, and r=.50 is large).

**Authors’ response:** Reviewer 1 is right. The smallest significant correlation (i.e., 0.05) may be significant based on the important amount of data for the within-person level. However, correlations in Table 2a are at between-person level and therefore only include a sample size of 62, corresponding to the number of different participants.

In the new version of the manuscript, we adapted the description and interpretation of these analyses by adding the effect size. We indicated this in the data analyses section, now it reads (p. 10):

*“**According to the established rule [57], correlation coefficients of 0.10 are considered to have a small effect size; those of 0.30, a medium effect size; and those of 0.50 (and above), a large effect size.”*

In the results section, now it reads (pp. 12-13):

*“**At the between-person level (Table 2a), the intensity of positive and negative emotions was negatively correlated (large effect size). In addition, we observed that differentiation between positive emotions and differentiation between negative emotions were positively correlated (medium effect size) with each other. Higher differentiation of positive emotions was related to lower intensity of negative emotions (medium effect size). More adjustment problems were related to lower intensity of positive emotions and higher intensity of negative ones (both large effect sizes). The use of non-adaptive emotion regulation strategies was related to higher intensity of negative emotions (medium effect size), lower intensity of positive emotions (medium effect size), and lower differentiation of both positive (small effect size) and negative emotions (small effect size). A higher level of self-control was related to higher intensity of positive emotions (medium effect size) and less intensity of negative emotions (medium effect size) and to more differentiation between positive emotions (effect size below small cut off). Moreover, age was positively correlated with lower differentiation of negative emotions (medium effect size). SES was not related to any emotional features. Higher verbal IQ was associated with lower intensity of negative emotions (effect size below small cut off) and higher differentiation between both positive and negative emotions (effect size below small cut off). Puberty was positively associated with the intensity of negative emotions and negatively associated with the intensity of positive emotions and differentiation between both positive and negative emotions (small effect size).*

*-- INSERT TABLE 2 ABOUT HERE --*

*At the within-person level (controlling for between-person level, Table 2b), the momentary intensity of positive emotions was negatively related to the momentary intensity of negative emotions (medium effect size). In addition, differentiation between positive emotions and differentiation between negative emotions were positively correlated with each other (medium effect size). Time (across the 9 days) was related to lower momentary intensity of negative emotions, higher momentary differentiation of both positive and negative emotions, fewer momentary anger ruminations, and higher momentary self-control (all small effect sizes). Within a day (across the four assessments), adolescents had higher momentary intensity of positive emotions and lower momentary differentiation of positive emotions (both very small effect sizes). Higher differentiation of positive and negative emotions was associated with higher momentary intensity of positive emotions and lower momentary intensity of negative emotions (medium to large effect size). Anger rumination was negatively correlated with momentary positive emotions and positively with the intensity of negative emotions (small effect size). Self-control was negatively correlated with the momentary intensity of negative emotions (medium effect size), whereas it was positively correlated with the momentary positive intensity of emotions (medium effect size) and differentiation of positive and negative emotions (small effect sizes).”*

The abstract mentions "higher differentiation between negative emotions" in an external context, but the multilevel models computed on differentiation scores were "not reliable or significant and therefore not interpretable" (p. 13). This inconsistency needs to be resolved - you can't claim a finding in the abstract that you later state wasn't reliable.

**Authors’ response:** It seems there is a misunderstanding here. Higher differentiation between negative emotions refers to the results of the MANOVA, thus, addressing the differences in differentiation between emotions related to the context. The multilevel model refers to the model trying to understand the variances in differentiation scores. To avoid such misunderstanding, we rewrote the abstract accordingly. Now it reads:

*“**The dynamics of emotions (e.g., emotion intensity and differentiation) are pivotal for adolescents’ mental health. This study examined how psychological characteristics and the social environment influence the dynamics of six emotions in male adolescents during their daily lives. Sixty-two male adolescents (aged between 12 to 17 years, sufficiently fluent in French, and having access to a smartphone) with varying degrees of adjustment problems (assessed through the youth self-report questionnaire) participated in an experience sampling study (4x/day over 9 days, excluding weekends). Greater adjustment problems, were associated with fewer positive emotions, while greater use of non-adaptive emotion regulation was linked to more negative emotions. Being alone was linked to lower intensity of positive emotions and higher intensity of negative ones. Higher momentary self-control was related to higher intensity of positive emotions and lower intensity of negative ones. This study provides novel insights into the dynamics of male adolescents’ emotional experiences by enhancing the understanding of emotions’ contextual anchoring combined with psychological characteristics.*

*”*

There appears to be a disconnect between your research question and your conclusions. You initially aimed to examine "how psychological characteristics and the social environment influence the dynamics of emotions" but many conclusions stretch beyond what the data support, especially regarding causal inferences.

**Authors’ response:** Accordingly, we re-read again carefully the manuscript to better align the research question and the conclusions, and to avoid making conclusions that are not supported by our data and analyses. The conclusion part now reads as follows (p.19):

*“In conclusion, we observed associations at the trait level between adjustment problems, emotion regulation, and emotional experience, while self-control appeared to play a more specific role in real-time (i.e., state level) emotional dynamics. Finally, we observed specific developmental trends. In addition, our results highlight the crucial importance of the social environment and, in particular, the presence of others in the construction of the male adolescent identity, which underlies the dynamics of emotion. Therefore, our study provides important insights for a better understanding of the dynamics of emotion in the daily life of male adolescents.”*

Additional Issues:

Sample limitations need more acknowledgment. The exclusion of female participants severely limits generalizability, especially since you note gender differences in emotion dynamics. The age range of 12-17 is also quite broad considering the rapid developmental changes in this period.

**Authors’ response:** Indeed, the sample represent a limitation of the study. The results are not directly generalizable to females, and we acknowledge this in the current form of the manuscript. Regarding the included age range of 12-17, we added more information about this in the limitations section. Now it reads (p. 18):

*“This study suffers from several limitations that should be mentioned and that limit the generalizability of the results. Our sample, rather small, was solely composed of male adolescents with a relatively wide age range. Further studies may be conducted to generalize our results to more diverse populations in terms of gender and study more specifically the role of age.”*

The abstract is missing crucial methodological details. It states "62 male adolescents with varying degrees of adjustment problems," but doesn't clarify how these problems were measured or the inclusion criteria.

**Authors’ response:** Accordingly, we added this information in the Abstract. Now it reads:

*“The dynamics of emotions (e.g., emotion intensity and differentiation are pivotal for adolescents’ mental health. This study examined how psychological characteristics and the social environment influence the dynamics of emotions in male adolescents during their daily lives. Sixty-two male adolescents (aged between 12 to 17 years, sufficiently fluent in French, and having access to a smartphone) with varying degrees of adjustment problems (assessed through the youth self-report questionnaire) participated in an experience sampling study (4x/day over 9 days, excluding weekends). Greater adjustment problems were associated with fewer positive emotions, while greater use of non-adaptive emotion regulation was linked to more negative emotions. Being alone was linked to lower intensity of positive emotions and higher intensity of negative ones. Higher momentary self-control was related to higher intensity of positive emotions and lower intensity of negative ones. This study provides novel insights into the dynamics of male adolescents’ emotional experiences by enhancing the understanding of emotions’ contextual anchoring combined with psychological characteristics.”*

Your discussion on p.17-18 about Gross's process model of emotion regulation is speculative and goes beyond your data. You state "our findings align with Gross's process model" but then make numerous interpretive leaps without direct evidence.
The conclusion mentions "a stable influence of adjustment problems and emotion regulation" but your design can't determine stability since it's not longitudinal in the traditional sense.

**Authors’ response:** Thank you for rising this important point. Accordingly, we have now reworked this section to ensure that the speculations do not go beyond our data, and to avoid drawing conclusions that exceed what our design allows, thus, being more cautious about these interpretations. Now it reads (pp. 17-18):

*“**Taking a broader perspective, we can speculate about how the Gross’s process model of emotion regulation [e.g., 25, 70, 71], offers valuable insights on our results. Gross’s model provides a framework for understanding emotion regulation across different stages: situation selection, situation modification, attentional deployment, cognitive change, and response modulation. In the situation selection phase, based on our data which showed that being alone was associated with lower positive emotion intensity and higher negative emotion intensity), we might hypothesize that adolescents choose familiar environments or social interactions (especially with family and friends) to enhance positive emotions. This may imply that adolescents might select social contexts as a strategy for emotion regulation. Regarding the situation modification phase, our findings suggest that external contexts are linked to a higher differentiation of negative emotions, suggesting that altering one’s environment may be associated with emotional experiences. One hypothesis may be that adolescents might modify their surroundings to increase emotional granularity, either by seeking out new environments or by returning to familiar one to promote comfort. In the attentional deployment phase, our results on momentary self-control can be interpreted in the light of this framework. Indeed, higher momentary self-control was associated with greater positive emotion intensity and reduced negative emotion intensity. We can, thus, hypothesize that adolescents with better self-control may be more adept at directing their attention away from negative stimuli or toward positive aspects of their environment. At the cognitive change stage, our findings reveal that greater use of non-adaptive strategies (at the trait level) correlates with increased negative emotions, suggesting that adolescents who struggle with cognitive reappraisal or other adaptive strategies may experience more negative affect. This suggests that developing effective cognitive change techniques in emotion regulation might be helpful. By interpreting our results in the light of the Gross’s model of emotion regulation, we may gain some insights of how adolescents’ emotional experiences and regulation strategies correspond to different stages of emotion dynamics.”*

*”*

Your title suggests a comprehensive examination of emotion dynamics, but you only measured six emotions (three positive, three negative), which is a significant limitation that should be acknowledged more prominently.

**Authors’ response:** Accordingly, we adapted the title to more accurately reflect the study. Now the title reads:

*“Examining Specific Emotion Dynamics in Daily Life in Male Adolescents: An Experience Sampling Method Study”*

In addition, we stressed this in the limitations section, now it reads (p. 18):

“*Furthermore, we assessed a restricted range of emotions, limiting ourselves to six emotions. Other emotions not included in our assessment could play an important role in emotion dynamics.”*

The multilevel regression analyses (Table 3) show several significant coefficients that are very small in magnitude. For example, non-adaptive emotion regulation shows a significant coefficient of 0.37 for negative emotions - what does this mean in real-world terms? The practical significance of these findings is unclear.

**Authors’ response:** Thank you for raising this important question. We have now added more information to bring more clarity. In the data analyses section, now it reads (p.10):

“*In the multilevel regression model, we used unstandardized beta (or gamma) coefficients, which indicate the change in the outcome variable for a one-unit change in the explaining factors, expressed in the original units of measurement.”*

And in the results section, we added this specification for each significant effect. Now it reads (pp. 13-14):

*“At the between-person level, greater adjustment problems were related to lower positive emotions (decrease of 0.81 in positive emotions for each unit of increase in adjustment problems). Greater use of non-adaptive emotion regulation strategies was positively related to negative emotions (increase of 0.37 in negative emotions for each unit of increase in non-adaptive emotion regulation). A more advanced puberty stage was related to higher negative emotions (increase of 9.43 in negative emotions for each unit of increase in puberty), while older age was related to lower negative emotions (decrease of 3.79 in negative emotions for each unit of increase in age). At the within-person level, results revealed that being alone was associated with a lower intensity of positive emotions (decrease of 2.41 in positive emotions when being alone) and a higher intensity of negative emotions (increase of 2.40 in negative emotions when being alone). In the presence of an unfamiliar other, adolescents reported a lower intensity of positive emotions (decrease of 3.33 in positive emotions when being with unfamiliar others) and a higher intensity of negative emotions (increase of 4.59 in negative emotions when being with unfamiliar others). More anger rumination was related to lower intensity of positive emotions (decrease of 0.06 in positive emotions for each unit of increase in anger rumination). Higher states of self-control were related to higher intensity of positive emotions (increase of 0.28 in positive emotions for each unit of increase in self-control) and less intensity of negative emotions (decrease of 0.19 in negative emotions for each unit of increase in self-control).”*

The theoretical framing focuses heavily on maturation of the prefrontal cortex versus limbic system, but the study includes no neuroimaging measures. This creates a disconnect between your theoretical framework and methodology.

**Authors’ response:** According to Reviewer1’s remark, we have now omitted the unique sentence describing the neurobiological basis of behaviors related to emotions in the introduction section to avoid this impression of disconnection between the theoretical framework and our methodology.

There are several places where you make causal claims (e.g., "higher states of self-control were related to higher intensity of positive emotions and less intensity of negative ones") when your design only supports correlational relationships.

**Authors’ response:** We thanks Reviewer1 for this important remark. From our point of view, “*being related”* does not imply a direction or causal relationship, which we indeed cannot state given the design of the current study. We stressed this aspect in the limitations of the study. Additionally, we have now re-read carefully the manuscript to avoid the use of wording implying causal relationships.

The limitations section on p.17-18 doesn't address the issue of multiple statistical tests without proper correction, which increases the risk of Type I errors.

**Authors’ response:** Reviewer 1 is right to point out that we used multiple tests, especially in the correlational analysis (preliminary analyses). However, for the main analyses, we referred to the MANOVA and to 2 multilevel models. Thus, our main results are based on 3 different models. By consequence, we adjusted the level of significance of the p-value to (.05/3) and added this information in the manuscript. This will allow us to avoid Type I error. Now it reads (p. 10):

*“The main analyses referring to three models, the significance for the p-value was set at .017 (.05/3) to avoid Type I error.”*

You state that data is "provided within the manuscript" but it's unclear if the raw data will be accessible to other researchers, which is increasingly an expectation for publication.

**Authors’ response:** As stated above in response to Journal Requirement, we have now specified this aspect.

Summary:
This manuscript examines an important topic but has significant issues with the interpretation of results, particularly regarding small correlation coefficients that achieve statistical significance but lack practical importance. The abstract and conclusions make claims that aren't fully supported by the data, and there's insufficient acknowledgment of the limitations of the study design for making causal inferences.

**Authors’ response:** Again, we want to thank Reviewer 1 for the relevant and thoughtful comments that helped us to enhance the readability of the manuscript. We do hope that we appropriately addressed the Reviewer 1’s concerns.

**Reviewer 2**

This manuscript addresses a relevant and timely topic in adolescent psychology using an experience sampling method (ESM), which is suitable for capturing emotion fluctuations in real-life settings. The study focuses specifically on male adolescents, offering potential insights into gender-specific emotional processes. While the paper contributes to the growing field of emotion dynamics, there are areas that limit its overall impact.

Strengths:
- The use of ESM enhances ecological validity.

- The research question is clearly stated and addresses a gap in current literature.
- The manuscript is generally well-structured and readable.

**Authors’ response:** We would like to thank the Reviewer 2 for this encouraging feedback as well as for her/his thoughtful comments that greatly helped us enhance the clarity of the manuscript. Below, you can find a point-by-point response to the Reviewer’s comments.

Limitations:
- The sample size is relatively modest, which may limit generalizability.

**Authors’ response:** Indeed, our sample is rather small. It refers to the pilot nature of the study, which was supported by a small grant lasting 1 year. We highlighted the limitation of the sample in the limitation section.

- Some statistical analyses and interpretations could benefit from further clarification or justification.

**Authors’ response:** As described above in response to similar concerns from Reviewer 1, we have now added more information about the analyses, their description and their interpretation.

- The discussion section could better connect findings to broader theoretical frameworks

**Authors’ response:** As Reviewer 1 requested as well, especially regarding the Gross’ model, we have better connected the findings to the theoretical frameworks (see above for Reviewer 1’s responses).
