# Supplementary material for: Examining specific emotion dynamics in daily life in male adolescents: An experience sampling method study
Source: PLOS Ment Health. 2026 Jan 7;3(1):e0000513. doi: 10.1371/journal.pmen.0000513 (PMC12798519; doi:10.1371/journal.pmen.0000513)
Supplement: Attachment — Submitted filename: R2R2_submitteddocx.docx [file pmen.0000513.s008.docx]

**To the Editor and the Reviewers**

We would like to thank the Editor and the Reviewers for their encouraging feedback and new comments on our manuscript. In gray are the comments of the Editor and Reviewers, we respond below in normal text and the *text in italics* are part from the manuscript. In the track change version of the manuscript, the changes are highlighted in yellow.

**Editor**

**Consent:** You stated that “each adolescent and their legal representative completed a written consent form”, but you did not clarify how comprehension was ensured and whether assent was obtained separately from minors. Revise and clearly state these; comprehension and assent for minors

**Authors response:** Accordingly, the standardized procedure was described as follows:

*“Participants first receive oral information about the study, followed by written information for review. Written informed consent is then obtained from each participant before any study-specific procedure. The same process is applied to parents or legal representatives to ensure full understanding and agreement.”*

**Data Sharing:** Your current data availability statement does not comply with the PLOS Data Policy. It is not acceptable for the corresponding author to be the sole gatekeeper of the data. If some participants did not consent to data reuse, the minimal dataset from participants who did consent should still be shared. Publish your dataset in a public repository after removing the identifiers. You can use Harvard Dataverse or Mendeley or any other and show the digital object indentifier (DOI) of the dataset.

**Authors response:** Accordingly, we uploaded the minimal data set on Zenodo (DOI: 10.5281/zenodo.17341614). We adapted this information in the manuscript. Now it reads.

#### **“Availability of data and materials**

*Some participants did not consent on the re-use of their data, thus we are unable to share the whole dataset. However, we shared the minimal dataset which is available on zenodo (DOI:10.5281/zenodo.17341614).”*

**Reviewer #3**

An interesting study with an important aim to explore the effects of social environment and individual psychological characteristics on adolescent mental health and development. The vital age range of 12 to 17 years was chosen for this research study group. This is a wise choice because it is a stage of increased development of social intelligence, brain function, and reasoning, as well as psychological changes, which ultimately shape adult behaviors in male adolescents. The authors focused on 2 main features: emotional intensity and emotional granulation. They highlighted previous study reports on emotion regulation and further explained how these are categorized as adaptive and non-adaptive, with an emphasis on the positive and negative effects of utilizing either emotional category. They further explained how environment can contribute to fostering stronger positive emotions and adolescents e.g. types of social environment and the presence of others in the environment. Emphasis was placed on how there has been a lack of previous studies which addressed this vital state of adolescent development. Personally, as a clinician and study reviewer, I find it rather unfortunate because it is common knowledge that adolescents who learn to appropriately regulate their emotions by managing their stress and frustrations tend to develop better adult coping mechanisms. The authors employ the EMS (experienced sample method) for real-time emotional measurements in natural environments, with a focus on male subjects. The study was authorized by an Ethics Committee, and written consent was obtained from the study participants and their legal representatives. Study participants' exclusion was well detailed (diagnosis of mental health disorder and autism spectrum disorders). The various study measures and how these measures were obtained were well detailed. The authors presented well-structured statistical results with correlation analysis. This is a very important study that further substantiates the common knowledge that adolescent emotional experiences, characteristics, and environmental exposure contribute to the views that adolescent males have of themselves. Positive emotional States and a supportive environment tend to foster stronger self-esteem, while non-adaptive emotions, such as rumination, dramatization, and self-blaming, can negatively affect adult self-concept and resilience. This is an important study worthy of publication as an educational resource for identifying and further substantiating how the emotional states of adolescent males and social environment play a vital role in influencing personality development, relationship building, and coping skills in both young and older male adults.

**Authors response :** Thank you for this summary and encouraging feedback.

Minor revision suggestion: the last paragraph on page 16 may need a minor adjustment. It states that
“Moreover, our results highlighted that when adolescents are NOT in a familiar environment, they experience more positive emotions and fewer negative ones and are better able to differentiate between negative emotions”

The word …. “NOT”, in the first sentence of that paragraph should be removed so that the study conclusion will better correlate with the study details.

**Authors response:** We think there is a misunderstanding from the Reviewer. The “NOT” describes appropriately the results described in pp 10-11. Thus, we did not omit it.

Thank you so much.

**Authors response:** Thank to you.

**Reviewer #4:**

The revised version of your manuscript shows clear improvement. You have appropriately adjusted the title, clarified some methodological issues, and moderated causal interpretations. The abstract is more consistent with the reported results, and the inclusion of effect size interpretations is welcome. These steps strengthen the manuscript.
**Authors response:** Thank you for this positive feedback.

However, some important concerns remain before the paper can be considered for publication. These mainly relate to ethical transparency and data availability.

1. You state that “each adolescent and their legal representative completed a written consent form.”. Detail how comprehension of the study was ensured and whether any incentives were provided.

**Authors response :** Accordingly, we described in more details the consent procedure. Now it reads :

*“Participants first receive oral information about the study, followed by written information for review. Written informed consent is then obtained from each participant before any study-specific procedure. The same process is applied to parents or legal representatives to ensure full understanding and agreement.”*

2. The current statement does not fully comply with PLOS requirements. While you mention that raw data cannot be fully shared due to ethical restrictions, the policy requires either:

(a) Minimal Data Set or (b) a clear process through which a data access committee or institutional body can grant access under restrictions. Please note it is not acceptable for an author to be the sole named individual responsible for ensuring data access. Read PLOS Data policy.

Merely stating that the corresponding author can be contacted is insufficient. Please specify exactly which data are publicly available, what is restricted, why, and how researchers may formally request access.

**Authors response:** Accordingly, we uploaded the minimal data set on Zenodo (DOI:10.5281/zenodo.17341614). We adapted this information in the manuscript. Now it reads.

#### **“Availability of data and materials**

*Some participants did not consent on the re-use of their data, thus we are unable to share the whole dataset. However, we shared the minimal dataset which is available on zenodo (DOI:10.5281/zenodo.17341614).”*
